# Mediterranean dietary pattern and cancer risk in the EPIC cohort

**DOI:** 10.1038/bjc.2011.106

**Published:** 2011-04-05

**Authors:** E Couto, P Boffetta, P Lagiou, P Ferrari, G Buckland, K Overvad, C C Dahm, A Tjønneland, A Olsen, F Clavel-Chapelon, M-C Boutron-Ruault, V Cottet, D Trichopoulos, A Naska, V Benetou, R Kaaks, S Rohrmann, H Boeing, A von Ruesten, S Panico, V Pala, P Vineis, D Palli, R Tumino, A May, P H Peeters, H B Bueno-de-Mesquita, F L Büchner, E Lund, G Skeie, D Engeset, C A Gonzalez, C Navarro, L Rodríguez, M-J Sánchez, P Amiano, A Barricarte, G Hallmans, I Johansson, J Manjer, E Wirfärt, N E Allen, F Crowe, K-T Khaw, N Wareham, A Moskal, N Slimani, M Jenab, D Romaguera, T Mouw, T Norat, E Riboli, A Trichopoulou

**Affiliations:** 1 International Agency for Research on Cancer, Lyon, France; 2 Department of Nutrition, University of Oslo, Oslo, Norway; 3 The Tisch Cancer Institute, Mount Sinai School of Medicine, One Gustave L Levy Place, New York, NY 10029, USA; 4 Hellenic Health Foundation, Athens, Greece; 5 WHO Collaborating Center for Food and Nutrition Policies, Department of Hygiene, Epidemiology and Medical Statistics, University of Athens Medical School, Athens, Greece; 6 Data Collection and Exposure Unit, European Food Safety Authority, Parma, Italy; 7 Unit of Nutrition, Environment and Cancer, Cancer Epidemiology Research Programme, Catalan Institute of Oncology (IDIBELL), Barcelona, Spain; 8 Department of Epidemiology, School of Public Health, Aarhus University, Aarhus, Denmark; 9 Department of Clinical Epidemiology, Aarhus University Hospital, Aalborg, Denmark; 10 Department of Diet, Cancer and Health, Institute of Cancer Epidemiology, Danish Cancer Society, Copenhagen, Denmark; 11 Inserm, Centre for Research in Epidemiology and Population Health, U1018, Institut Gustave Roussy, Villejuif F-94805, France; 12 Paris South University, UMRS 1018, Villejuif F-94805, France; 13 Department of Epidemiology, Harvard School of Public Health, Boston, MA, USA; 14 Bureau of Epidemiologic Research, Academy of Athens, Athens, Greece; 15 Division of Cancer Epidemiology, German Cancer Research Center, Heidelberg, Germany; 16 Department of Epidemiology, German Institute of Human Nutrition, Potsdam-Rehbrücke, Germany; 17 Department of Clinical and Experimental Medicine, Federico II University, Naples, Italy; 18 Nutritional Epidemiology Unit, Fondazione IRCSS Istituto Nazionale dei Tumori, Milan, Italy; 19 Department of Epidemiology and Biostatistics, School of Public Health, Imperial College, London, UK; 20 Department of Epidemiology and Life Sciences, Institute for Scientific Interchange Foundation, Torino, Italy; 21 Molecular and Nutritional Epidemiology Unit, Cancer Research and Prevention Institute–ISPO, Florence, Italy; 22 Cancer Registry and Histopathology Unit, ‘Civile MP Arezzo’, ASP 7, Ragusa, Italy; 23 Julius Center for Health Sciences and Primary Care, University Medical Center, Utrecht, The Netherlands; 24 National Institute for Public Health and the Environment (RIVM), Bilthoven, The Netherlands; 25 Department of Gastroenterology and Hepatology, University Medical Centre Utrecht, Utrecht, The Netherlands; 26 Department of Epidemiology, Biostatistics and HTA, Radboud University Nijmegen Medical Centre, Nijmegen, The Netherlands; 27 Department of Community Medicine, University of Tromsø, Tromsø, Norway; 28 Consortium for Biomedical Research in Epidemiology and Public Health, CIBER de Epidemiología y Salud Pública, Barcelona, Spain; 29 Department of Epidemiology, Murcia Regional Health Council, Murcia, Spain; 30 Public Health and Participation Directorate, Health and Health Care Services Council, Asturias, Spain; 31 Granada Cancer Registry, Andalusian School of Public Health, Granada, Spain; 32 Public Health Division of Gipuzkoa, Basque Government, San Sebastian, Spain; 33 Service of Epidemiology, Prevention and Health Promotion, Public Health Institute of Navarra, Pamplona, Spain; 34 Department of Public Health and Clinical Medicine, Umeå University, Umeå, Sweden; 35 Department of Odontology, Umeå University, Umeå, Sweden; 36 Department of Surgery, Skåne University Hospital, Lund University, Malmö, Sweden; 37 Department of Clinical Sciences in Malmö/Nutrition Epidemiology, Lund University, Malmö, Sweden; 38 Cancer Epidemiology Unit, Nuffield Department of Clinical Medicine, University of Oxford, Oxford, UK; 39 Department of Public Health and Primary Care, University of Cambridge, Cambridge, UK

**Keywords:** Mediterranean diet, dietary patterns, cancer risk, epidemiology

## Abstract

**Background:**

Although several studies have investigated the association of the Mediterranean diet with overall mortality or risk of specific cancers, data on overall cancer risk are sparse.

**Methods:**

We examined the association between adherence to Mediterranean dietary pattern and overall cancer risk using data from the European Prospective Investigation Into Cancer and nutrition, a multi-centre prospective cohort study including 142 605 men and 335 873. Adherence to Mediterranean diet was examined using a score (range: 0–9) considering the combined intake of fruits and nuts, vegetables, legumes, cereals, lipids, fish, dairy products, meat products, and alcohol. Association with cancer incidence was assessed through Cox regression modelling, controlling for potential confounders.

**Results:**

In all, 9669 incident cancers in men and 21 062 in women were identified. A lower overall cancer risk was found among individuals with greater adherence to Mediterranean diet (hazard ratio=0.96, 95% CI 0.95–0.98) for a two-point increment of the Mediterranean diet score. The apparent inverse association was stronger for smoking-related cancers than for cancers not known to be related to tobacco (*P* (heterogeneity)=0.008). In all, 4.7% of cancers among men and 2.4% in women would be avoided in this population if study subjects had a greater adherence to Mediterranean dietary pattern.

**Conclusion:**

Greater adherence to a Mediterranean dietary pattern could reduce overall cancer risk.

Since the early 1990s, growing evidence indicates that the Mediterranean diet, a concept first proposed by Keys in the mid-1980s (Keys *et al*, 1986), has a beneficial influence on health and longevity (Trichopoulou *et al*, 1995, 2005; Trichopoulou, 2004; Sofi *et al*, 2008, 2010). The Mediterranean diet reflects the dietary pattern prevalent in the olive growing areas of the Mediterranean region up to the 1960s (Trichopoulou, 2004). It is characterised by high intake of (i) vegetables, (ii) legumes, (iii) fruits and nuts, (iv) minimally processed cereals, (v) moderately high intake of fish, (vi) high intake of monounsaturated lipids coupled with low intake of saturated fat, (vii) low-to-moderate intake of dairies, (viii) low intake of meat products, and (ix) regular but moderate intake of alcohol (Trichopoulou *et al*, 1995). Adherence to a Mediterranean dietary pattern has been measured using a score with components reflecting intake of each dietary factor (Trichopoulou *et al*, 1995, 2005; Trichopoulou, 2004).

Investigating the association between adherence to Mediterranean diet and overall cancer risk would provide an integrated estimate of the potential beneficial effects of this habit on cancer burden. Although most studies have investigated the relationship between adherence to Mediterranean diet and overall mortality (Mitrou *et al*, 2007; Sofi *et al*, 2008, 2010) only a fairly small study from the Greek European Prospective Investigation into Cancer and Nutrition (EPIC) cohort (Benetou *et al*, 2008) has examined overall cancer incidence. Results of cohort studies on the association of adherence to Mediterranean diet with the risk of specific cancers have also been reported (Fung *et al*, 2006; Reedy *et al*, 2008; Cottet *et al*, 2009; Buckland *et al*, 2010; Trichopoulou *et al*, 2010).

In this report, we investigate the association between adherence to the Mediterranean dietary pattern and overall cancer risk within the EPIC study, a prospective cohort study including participants from 10 European countries.

## Materials and methods

### Study population and design

The EPIC study is a multi-centre prospective cohort study designed to investigate the relationship between nutrition and cancer. This study, described previously (Riboli *et al*, 2002), recruited ∼520 000 people mostly aged 25–70 years, between 1992 and 2000, in 23 centres located in Denmark, France, Germany, Greece, Italy, The Netherlands, Norway, Spain, Sweden, and the United Kingdom. Participants were mostly recruited from the general population. Exceptions were the French cohort (including female members of the health insurance for school and university employees), the Utrecht cohort in the Netherlands (recruiting women attending breast cancer screening), the Ragusa cohort in Italy (based on blood donors and their spouses), the Spanish cohorts (general population, blood donors, and civil servants) and the Oxford cohort in the United Kingdom, including mostly vegetarian and health-conscious volunteers. The cohorts of France, Norway, Utrecht, and Naples were restricted to women. Among individuals with complete exposure information, prevalent cases of cancer (23 633), subjects with incomplete follow-up information (9665), or with a ratio of energy intake *vs* energy expenditure in the top or bottom 1% (9672) were excluded. This left 142 605 men and 335 873 women followed for a median time of 8.7 years.

### Exposure assessment

At enrolment, questionnaires on lifestyle and other exposures were administered, and anthropometric measurements were obtained. Information on foods and beverages consumed during the year preceding enrolment was collected using instruments developed and validated within each centre (Margetts and Pietinen, 1997). Self- or interviewer-administered food frequency questionnaires, as well as 7- or 14-day record diaries (UK, and one Swedish centre) and diet history questionnaires (Spain) were used.

### The Mediterranean diet score

Adherence to a Mediterranean dietary pattern was assessed using the 9-Unit dietary score proposed by Trichopoulou *et al* (1995), including fruits and nuts, vegetables, legumes, cereals, lipids, fish, dairy products, meat products, and alcohol. We used a variant of this score (Trichopoulou *et al*, 2005), in which lipid intake was assessed by calculating the ratio of unsaturated (the sum of monounsaturated and polyunsaturated lipids) to saturated lipids, to allow for the low consumption of olive oil-derived monounsaturated lipids in non-Mediterranean countries (Trichopoulou, 2004). A value of 0 or 1 was assigned to each component of the score as follows: for components that are more consumed in Mediterranean countries (vegetables, legumes, fruits and nuts, cereals, fish, and a high ratio of unsaturated to saturated lipids), persons whose consumption was below or equal to the country sex-specific median were assigned a value of 0, and 1 otherwise. For components traditionally less consumed in Mediterranean countries (dairy, meat, and meat products), persons whose consumption was below the country- and sex-specific median were assigned a value of 1, and 0 otherwise. A value of 1 was given to persons consuming a moderate amount of alcohol (i.e., 10 to <50 g per day of ethanol for men and 5 to <25 g per day for women). For consumption of other quantities of alcohol a value of 0 was assigned. No information on legume consumption was available for the Norwegian cohort but sensitivity analyses, excluding Norway, did not influence the overall results. High scores correspond to high adherence to the Mediterranean dietary pattern (score's range: 0–9).

The score was also calculated using an alternative, more quantitative, method described in the appendix. Obtained results were very similar (data not shown).

### Outcomes

In most countries (Denmark, Italy, The Netherlands, Norway, Spain, Sweden, and the UK), incident cancers were identified through a linkage with population-based cancer registries. Cancer cases were also ascertained by active follow-up, through a health insurance company (France), or direct contacts with study subjects, their doctors, or their next of kin (France, Germany, and Greece). The end of follow-up ranged from December 2002 to December 2005, depending on centre. Cancers were classified using the International Classification of Diseases, 10th Revision (http://www.who.int/classifications /apps/icd/icd10online/), and the International Classification of Diseases for Oncology (second revision; Constance and Van Holten, 1990). Non-melanoma skin cancers and second primary cancers were excluded.

### Statistical analysis

The hazard ratio (HR) for overall cancer by the Mediterranean diet score was estimated using Cox regression analyses, with age as offset variable, through a categorical approach (a score equal to 0–3, 4, 5, and 6–9, dividing the population in four approximately equal groups), and a continuous one (a two-point increment in the score). Analyses were stratified by centre and sex, and adjusted for height (continuous), body mass index (BMI) (continuous), physical activity (categorical: inactive, moderately inactive, moderately active, active, missing), education (categorical: none, primary school, technical/professional school, secondary school, higher education, not specified), total energy intake (continuous), and tobacco smoking, categorical, comprising current amount of smoking (1–14, 15–24, or ⩾25 per day cigarettes), duration of smoking for current smokers in 10-years categories (⩽10, 11–20, 21–30, 31–40, 41–50, or >50 years), time since quitting (⩽10, >10–20, >20 years), smoking of pipe or cigar, occasional smoking, and missing smoking information. For women, the analyses were also adjusted for age at menarche (continuous), pregnancy (categorical: never, ever), oral contraceptives and hormone replacement therapy use (categorical: never, ever), and menopausal status (categorical: premenopausal, postmenopausal, perimenopausal, surgical postmenopausal). To explore possible effect modification by tobacco smoking and alcohol drinking, analyses were further stratified by these two habits, using the following categories: never, former, and current smokers; drinkers of <5 g per day of ethanol, 5 to <25, and ⩾25 g per day for women; and <10, 10 to <50, and ⩾50 g per day for men. We also examined the association between adherence to the Mediterranean dietary pattern and cancers known to be associated with tobacco smoking (i.e., lung, kidney, upper aero-digestive tract, stomach, pancreas, bladder, liver, and colorectal; IARC, 2004; Secretan *et al*, 2009), and to alcohol drinking (i.e., upper aero-digestive tract, breast, liver, and colorectal cancers; Secretan *et al*, 2009).

Attributable fractions were estimated based on the number of cancers that would be prevented if the whole population shifted its diet to that observed in the highest category of the score (⩾6; Hanley, 2001).

## Results

Overall, 9669 cases of cancer were identified in men and 21 062 in women. A higher adherence to the Mediterranean diet was observed in participants with higher education level, more physically active and among never and former smokers (Table 1). However, these results are not mutually adjusted and serve descriptive purposes only.

Adherence to the Mediterranean dietary pattern was significantly associated with a reduction in cancer risk (Table 2). The HR of all cancers associated with a two-point increment of the Mediterranean score was 0.96 (95% CI: 0.95–0.98) overall, 0.97 (95% CI 0.95–1.00) in men, and 0.96 (95% CI 0.95–0.98) in women. No differences were found between Southern and Northern European countries, with HRs of 0.97 (95% CI 0.95–0.99), and 0.96 (95% CI 0.94–0.98), respectively, for both genders combined. The results reached the conventional level of statistical significance only in Greece and Denmark.

A dose–response relation was observed; with lower risks observed with increasing adherence to Mediterranean pattern (Table 3). Overall the category-specific HRs were 0.96 (95% CI 0.93–0.99), 0.92 (95% CI 0.89–0.95), and 0.93 (95% CI 0.90–0.96) for scores of 4, 5, and 6–9 compared with 0–3 (*P* for linear trend=0.00001).

When considering overall cancer risk and the nine food groups considered in the Mediterranean diet score, an apparent protective effect of fruits and nuts, vegetables, cereals, and of a high ratio of unsaturated to saturated lipids was observed (Table 4). Increased meat consumption was associated with increased risk of cancer. In comparison with moderate drinkers (10 to <50 g per day for men and 5 to <25 g per day for women), the HR for all cancers was 1.03 (95% CI 1.00–1.06) for non- or light drinkers (<10 g per day of ethanol for men and <5g per day for women) and 1.12 (95% CI 1.07–1.16) for heavy drinkers (⩾50 g per day for men and ⩾25 g per day for women).

When stratifying by tobacco smoking, the apparent protection conveyed by adherence to the Mediterranean dietary pattern was somewhat stronger among current smokers than among never smokers. The HRs for a two-point increment in the score were 0.98 (95% CI 0.96–1.00) among never smokers, 0.96 (95% CI 0.93–0.98) among former smokers, and 0.94 (95% CI 0.91–0.97) among current smokers (*P* (heterogeneity)=0.07; Table 5). The apparent protective effect of adhering to the Mediterranean dietary pattern was more pronounced among cancers known to be related to smoking. Per two-point increment in the Mediterranean diet score, the HR for tobacco-related cancers was 0.92 (95% CI 0.89–0.94); that for cancers not known to be related to tobacco 0.98 (95% CI 0.96–1.01; *P* (heterogeneity)=0.008). There was no difference in the apparent effect of adherence to the Mediterranean dietary pattern on overall cancer risk when different strata of alcohol consumption, or alcohol-related and not related cancers were considered (results not shown).

When translating the categorical relative risk measures into attributable fractions, we found that 4.7% of cancers in men and 2.4% of cancers in women would be avoided if all study subjects shifted in the highest category of the Mediterranean diet score (⩾6 points).

## Discussion

In this study, adherence to the Mediterranean dietary pattern was associated with lower cancer risk. The association was moderately strong, but about 4.7% of cancers in men and 2.4% of cancers in women would have been prevented in this population if it shifted to the category with the highest adherence to a Mediterranean dietary pattern (score ⩾6), adhered to by less than 50% of this population (Table 1). It should be noticed that an attributable fraction of 3–5% is comparable with that of other established causes of cancer, notably obesity, and alcohol drinking (Bergstrom *et al*, 2001; Boffetta *et al*, 2006).

Several studies have reported a beneficial effect of the Mediterranean diet on health and longevity (Trichopoulou *et al*, 1995, 2005; Trichopoulou, 2004; Sofi *et al*, 2008, 2010). In 2008, a meta-analysis of six cohort studies of adherence to Mediterranean diet and cancer reported an overall relative risk of 0.94 (95% CI: 0.92–0.96) for a two-point increase in the score (Sofi *et al*, 2008, 2010). Our study has some advantages over this meta-analysis. First, it includes more than three times as many cases of cancer. Second, it is based on a series of incident cases, whereas the meta-analysis (Sofi *et al*, 2008, 2010) included four studies of cancer mortality, two studies investigating specific cancer sites incidence (breast and gastric cancers), and one of total cancer incidence. Third, it is based on a standard definition of adherence to Mediterranean dietary pattern for all contributing centres. Lastly, it relied on a very detailed adjustment for potential confounders, notably tobacco smoking. Results from case–control studies on adherence to Mediterranean diet in relation to specific cancer sites have been reported for lung cancer (Fortes *et al*, 2003), cutaneous melanoma (Fortes *et al*, 2008), colorectal adenoma (Dixon *et al*, 2007), and upper aero-digestive tract cancers (Bosetti *et al*, 2003). In general, they suggested a more pronounced protective effect of this diet in comparison with our study. This could be because of the information bias in retrospective investigations or to real differences because of the geographic idiosyncrasies in the association between Mediterranean diet and cancer risk. Compared with our study, the NIH-AARP Diet and Health cohort study of total cancer mortality observed a slightly stronger protective effect for adherence to Mediterranean diet (Mitrou *et al*, 2007). Our study had a substantially larger number of cancer cases, and relied on cancer incidence rather than mortality as outcome. However, although in most countries (Denmark, Italy, The Netherlands, Norway, Spain, Sweden, and the UK), cancer cases were identified through linkage with population-based cancer registries, outcome misclassification cannot be ruled out in countries using other methods to identify cancer cases.

The association between adherence to the Mediterranean dietary pattern and cancer risk appeared to be stronger for smokers, and cancers known to be related to tobacco smoking. A lower risk of lung cancer with greater adherence to Mediterranean diet has previously been reported (Fortes *et al*, 2003). Although we used detailed adjustment for current amount of smoking, duration of smoking for current smokers, time since quitting for former smokers, smoking of pipe or cigar, and occasional smoking, we cannot exclude residual confounding effect. Other adjustments for smoking were used and yielded similar results (results not shown). When considering cancers not known to be related to smoking, the protective effect of adhering to Mediterranean dietary was not statistically significant. It is possible that residual confounding by smoking explains some the association between conformity to Mediterranean pattern and overall and smoking related cancers. However, exclusion of terms for tobacco smoking from the model yielded a HR of 0.95 (95% CI 0.94–0.97) per two-point increment in the Mediterranean diet score compared with 0.96 (95% CI 0.95–0.98) after detailed adjustment for smoking. This suggests that the apparent modest protective effect of the Mediterranean diet on overall cancer risk is real, and unlikely to be because of the residual confounding by smoking. Similarly, in the large NIH-AARP Diet and Health study, detailed adjustment for smoking had only a modest effect on the association between adherence to Mediterranean diet and cancer mortality (Mitrou *et al*, 2007). Given the difference in baseline cancer risk between smokers and non-smokers, the difference in the effect of adherence to Mediterranean diet on an absolute scale is likely to be larger than indicated by a relative scale. The predominant effect on smoking-related cancers would be consistent with the anti-oxidant properties of the Mediterranean diet (Owen *et al*, 2000), as cigarette smoke contains free radicals and induces oxidative damage (IARC, 2004). Of note, in a study conducted within the Greek EPIC cohort, in which a particularly strong inverse association between adherence to the Mediterranean diet and cancer risk was evident (Greek sub-cohort results in Table 2), the association was stronger for smoking unrelated cancers (Benetou *et al*, 2008). Residual confounding by other variables is possible. However, the statistical model used controlled for a large number of potential confounders such as education, BMI, total energy intake, physical activity further to smoking. As dietary questionnaires were used to measure conformity to Mediterranean pattern, measurement error is possible. However, questionnaires used were validated within each centre (Margetts and Pietinen, 1997). Furthermore, it is likely that any existing measurement error is randomly distributed between cancer cases and non-cases due to, in particular, the prospective nature of the EPIC study. Such random measurement error would bias our results towards the null. One limitation of this study is that measurement of exposure and possible confounders was carried out only once. Diet and lifestyle information was only ascertained at enrolment measuring habits over the 12-months period preceding the enrolment. It is possible that changes in diet and possible confounders have occurred during the follow-up time of this study (median time: 8.7 years).

Using the modified score (Trichopoulou *et al*, 2005) to investigate the effect of Mediterranean diet on cancer risk represents a compromise between the need to identify the beneficial aspect of this dietary pattern and the need to assess it in non-Mediterranean countries. Some important features of the Mediterranean diet are not captured by the score, such as the use of wine during meals as main source of alcohol and of olive oil as main source of unsaturated fat. The extent to which these features are important in mediating the effects of the Mediterranean diet on health is not easily quantifiable. The HR estimates calculated in a prospective study, however, would probably be biased towards the null on account of misclassification in the scoring assessment. The lack of a systematic difference in results between Mediterranean and non-Mediterranean countries (Table 2) argues in favour of the ability of the modified score (Trichopoulou *et al*, 2005) to adequately characterise the biologically relevant features of the Mediterranean diet. To construct the score, country-, and sex-specific medians of considered food groups were calculated. However, when using study-wide sex-specific medians, obtained results were similar. A recent analysis of the Greek EPIC cohort has identified moderate alcohol drinking, followed by low consumption of meat, and high consumption of vegetables, fruits and nuts, olive oil, and legumes as the most important components for the effect of the Mediterranean diet on overall survival (Trichopoulou *et al*, 2009). To evaluate whether the protective effect observed in this analysis was predominantly because of a specific component of the score, we performed sensitivity analyses eliminating one component at a time from the score, as well as alcohol and an another component at a time. These analyses did not suggest a predominant effect of a specific component (results not shown) and support the hypothesis that the beneficial effect is because of the combined effect of a range of nutrient and non-nutrient components, provided by a diet rich in antioxidants, fiber and phytochemicals and with a favourable fatty acid profile.

Potential biological interactions among different nutrients within the components of the Mediterranean diet score may be difficult to detect because of power limitations. The Mediterranean diet scoring approach (Trichopoulou *et al*, 1995) accommodates likely nutritional confounding, and captures possible effect modification among foods (Jacques and Tucker, 2001). One limitation of the score is that it gives equal weight to its components and only considers whether consumption of each component is above or below a certain threshold. The score was also calculated taking into account the absolute consumption of each component (see Appendix). The results of this method did not provide additional insights. We therefore relied on the results based on the standard scoring approach, which is simpler and intuitively appealing.

The EPIC study offers a unique possibility to investigate the relationship between adherence to Mediterranean dietary pattern and overall cancer risk. This is the case not only because of its large sample size, but also because information on diet and potential confounding factors was gathered prospectively, and is therefore unaffected by recall bias. Furthermore, one specific advantage of this study is the inclusion of a geographically diverse population including Mediterranean and non-Mediterranean countries. The diet of the participants is, therefore, heterogeneous and this study allows to investigate the association between conformity to Mediterranean dietary pattern and overall cancer risk both in Mediterranean and non-Mediterranean countries, which, to our knowledge, has not been carried out before. No difference in cancer risk associated with adherence to a Mediterranean dietary pattern was found between Mediterranean and non-Mediterranean countries, suggesting that the beneficial effect of the Mediterranean dietary pattern on health is also relevant to non-Mediterranean populations. It should be noted, however, that these results do not exclude the possibility that other diets, such as the traditional Dutch diet (Waijers *et al*, 2006), might also potentially positively impact not only on total mortality but also on cancer risk.

Our results indicate that higher adherence to a Mediterranean dietary pattern is associated with a reduction in the risk of cancer in Mediterranean and non-Mediterranean countries, with a somewhat stronger protective effect among smokers and against tobacco-related cancers. Promoting the Mediterranean diet might therefore contribute to cancer prevention, in addition to cardiovascular disease prevention (Fung *et al*, 2009). This also applies to Mediterranean countries, in which diet is shifting away from the traditional pattern and increasingly includes meat and fat of animal origin (Trichopoulos and Lagiou, 2004; Balanza *et al*, 2007).

## Figures and Tables

**Table 1:** Baseline characteristics according to categories of the Mediterranean score in the EPIC study by sex

	**Men**				**Women**			
	**Score: 0–3**	**Score: 4**	**Score: 5**	**Score: 6–9**	**Score: 0–3**	**Score: 4**	**Score: 5**	**Score: 6–9**
*N* (%)	43 161 (30.3)	30 770 (21.6)	29 766 (20.9)	38 908 (27.3)	110 891 (33.0)	75 166 (22.4)	69 906 (20.8)	79 910 (23.8)
								
Age at recruitment (years; mean±s.d.)	52.1 (10.4)	52.1 (10.1)	52.3 (10.0)	52.2 (9.9)	51.2 (9.6)	50.9 (9.7)	50.7 (9.8)	50.2 (10.1)
								
Height (cm; mean±s.d.)	174.5 (7.4)	174.6 (7.3)	174.8 (7.3)	175.0 (7.3)	162.1 (6.7)	162.3 (6.7)	162.3 (6.7)	162.5 (6.7)
								
Body mass index (mean±s.d.)	26.5 (3.7)	26.5 (3.6)	26.5 (3.6)	26.4 (3.6)	25.1 (4.5)	25.0 (4.5)	24.9 (4.4)	24.7 (4.3)
								
Total energy intake (kcal; mean±s.d.)	2308.2 (669.2)	2398.7 (671.2)	2466.5 (681.2)	2529.3 (654.4)	1830.8 (510.7)	1919.3 (542.7)	1987.7 (550.5)	2076.0 (537.8)
								
*Education (N, %)*								
None or primary	15 966 (33.2)	10 667 (22.2)	9882 (20.6)	11 500 (23.9)	35 884 (32.4)	21 914 (29.1)	18 730 (26.8)	18 685 (23.4)
Technical/professional	10 823 (31.5)	7507 (21.8)	7173 (20.9)	8884 (25.8)	25 099 (22.6)	16 249 (21.6)	14 910 (21.3)	15 910 (19.9)
Secondary or university	14 993 (26.6)	11 804 (20.9)	12 002 (21.3)	17 543 (31.1)	45 291 (40.8)	34 125 (45.4)	33 711 (48.2)	42 235 (52.8)
Missing	1379 (35.7)	792 (20.5)	709 (18.4)	981 (25.4)	4617 (4.2)	2878 (3.8)	2555 (3.6)	3080 (3.8)
								
*Physical activity (N, %)*								
Inactive	7706 (29.4)	5825 (22.2)	5532 (21.1)	7165 (27.3)	14 399 (31.4)	10.338 (22.5)	9715 (21.2)	11 445 (24.9)
Moderately inactive	10 967 (29.3)	8076 (21.6)	7990 (21.3)	10 427 (27.8)	33 932 (31.9)	23 341 (21.9)	22 351 (21.0)	26 800 (25.2)
Moderately active	13 058 (28.1)	9777 (21.0)	10 014 (21.6)	13 589 (29.3)	34 825 (31.5)	24 227 (21.9)	23 369 (21.2)	27 987 (25.3)
Active	4735 (27.2)	3611 (20.7)	3789 (21.8)	5264 (30.2)	6437 (29.4)	4689 (21.4)	4689 (21.4)	6096 (27.8)
Missing	6695 (44.4)	3481 (23.1)	2441 (16.2)	2463 (16.3)	21 298 (41.6)	12 571 (24.5)	9782 (19.1)	7582 (14.8)
								
*Smoking status (N, %)*								
Never	13 999 (29.8)	10 023 (21.3)	9751 (20.8)	13 165 (28.0)	60 345 (32.3)	41 849 (22.4)	39 108 (20.9)	45 498 (24.4)
Former	13 920 (26.9)	10 922 (21.1)	11 240 (21.7)	15 668 (30.3)	22 719 (30.0)	16 587 (21.9)	16 458 (21.7)	20 017 (26.4)
Current	14 476 (34.5)	9402 (22.4)	8426 (20.1)	9632 (23.0)	25 071 (38.2)	14 873 (22.7)	12 764 (19.5)	12 749 (19.5)
Missing	766 (38.7)	423 (21.3)	349 (17.7)	443 (22.4)	2756 (35.2)	1857 (23.7)	1576 (20.1)	1646 (21.0)
								
*Geographical region (N, %)*								
Southern countries	11 741 (29.5)	8661 (21.8)	8489 (21.3)	10 879 (27.3)	44 648 (32.2)	30 809 (22.3)	29 167 (21.1)	33 798 (24.4)
Greece	3172 (29.9)	2268 (21.4)	2228 (21.0)	2933 (27.7)	5250 (35.0)	3327 (22.1)	3068 (20.4)	3374 (22.5)
Spain	4440 (29.3)	3339 (22.0)	3277 (21.6)	4096 (27.0)	8500 (34.2)	5677 (22.8)	5244 (21.1)	5436 (21.9)
Italy	4129 (29.5)	3054 (21.8)	2984 (21.3)	3850 (27.5)	10 390 (34.1)	6426 (21.1)	6091 (20.0)	7590 (24.9)
France					20 508 (30.1)	15 379 (22.6)	14 764 (21.7)	17 398 (25.6)
Northern countries	31 420 (30.5)	22 109 (21.5)	21 277 (20.7)	28 029 (27.3)	66 243 (33.5)	44 357 (22.5)	40 739 (20.6)	46 112 (23.3)
United Kingdom	7632 (33.4)	4484 (19.6)	4306 (18.8)	6454 (28.2)	16 587 (31.5)	10 466 (19.9)	10 393 (19.7)	15 218 (28.9)
The Netherlands	2633 (26.9)	2348 (24.0)	2285 (23.3)	2518 (25.7)	8210 (30.9)	6288 (23.7)	5785 (21.8)	6246 (23.5)
Germany	5539 (26.7)	4993 (23.1)	4871 (22.6)	6181 (28.6)	7857 (28.1)	6520 (23.4)	6412 (23.0)	7126 (25.5)
Sweden	7935 (35.6)	5022 (22.5)	4507 (20.2)	6181 (28.6)	8230 (31.2)	6504 (24.7)	5942 (22.5)	5704 (21.6)
Denmark	7681 (29.2)	5262 (20.0)	5308 (20.2)	8032 (30.6)	9382 (32.6)	6017 (20.9)	5779 (20.1)	7558 (26.3)
Norway					15.977 (45.3)	8562 (24.3)	6428 (18.2)	4260 (12.1)

**Table 2:** Hazard ratios for all cancers associated with a two-point increment of the Mediterranean diet score by EPIC participating countries

	**Men**		**Women**		**Both sexes**	
	**Cases**	**HR^a^** **(95% CI)**	**Cases**	**HR^b^ (95% CI)**	**Cases**	**HR^a^^,^^b^ (95% CI)**
*Southern countries*						
Greece	402	0.85 (0.76–0.96)	400	0.86 (0.76–0.97)	802	0.86 (0.79–0.94)
Spain	938	0.94 (0.86–1.01)	999	1.05 (0.97–1.14)	1937	0.99 (0.94–1.05)
Italy	735	0.97 (0.89–1.06)	1676	0.96 (0.91–1.02)	2411	0.97 (0.92–1.02)
France			6514	0.98 (0.95–1.01)	6514	0.98 (0.95–1.01)
Overall	2075	0.93 (0.88–0.99)	9589	0.98 (0.95–1.00)	11 537	0.97 (0.95–0.99)
						
*Northern countries*						
United Kingdom	1689	1.02 (0.97–1.08)	2907	0.96 (0.92–1.02)	4596	0.98 (0.95–1.02)
The Netherlands	311	0.93 (0.81–1.08)	1667	0.96 (0.90–1.02)	1978	0.96 (0.90–1.01)
Germany	1395	1.01 (0.94–1.08)	1299	0.94 (0.88–1.01)	2694	0.98 (0.93–1.02)
Sweden	2324	0.98 (0.93–1.04)	2286	0.96 (0.91–1.01)	4610	0.97 (0.93–1.01)
Denmark	1875	0.90 (0.86–0.95)	2161	0.93 (0.88–0.98)	4036	0.92 (0.89–0.95)
Norway			1153	0.98 (0.91–1.07)	1153	0.98 (0.91–1.07)
Overall	7594	0.97 (0.94–0.99)	11 473	0.95 (0.93–0.98)	19 067	0.96 (0.94–0.98)
						
*P* for heterogeneity between groups of countries		0.27		0.10		0.48
						
Overall	9669	0.97 (0.95–1.00)	21 062	0.96 (0.95–0.98)	30 731	0.96 (0.95–0.98)
Overall unadjusted^c^	9669	0.95 (0.92–0.97)	21 062	0.96 (0.95–0.98)	30 731	0.96 (0.94–0.97)
Overall without Greece^d^	9267	0.97 (0.95–1.00)	20 662	0.96 (0.95–0.98)	29 929	0.97 (0.95–0.98)

Abbreviations: CI=confidence interval; EPIC=European Prospective Investigation into Cancer and Nutrition; HR=hazard ratio.

a Stratified by centre and (for both sexes) sex, and adjusted for smoking status, duration of smoking, education, height, body mass index, total energy intake, physical activity.

b Adjusted for all the above variables, and age at menarche, parity, menopausal status, oral contraceptive and hormone therapy use.

c Analyses were only stratified by centre and (for both sexes) sex.

d Results for the Greek EPIC cohort were published previously (Benetou *et al*, 2008) and were excluded to present new results separately.

**Table 3:** Hazard ratios for all cancers associated with categories of the Mediterranean diet score

**Score**	**Cohort members**	**Cases**	**HR^a^ (95% CI)**
*Both sexes*			
0–3	154 052	10 349	1.00
4	105 936	6849	0.96 (0.93–0.99)
5	99 672	6225	0.92 (0.89–0.95)
6–9	118 818	7308	0.93 (0.90–0.96)
		*P* for trend=0.00001	
			
*Men*			
0–3	43 161	3044	1.00
4	30 770	2121	0.99 (0.93–1.04)
5	29 766	2049	0.97 (0.92–1.03)
6–9	38 908	2455	0.93 (0.88–0.99)
		*P* for trend=0.02	
			
*Women*			
0–3	110 891	7305	1.00
4	75 166	4728	0.95 (0.91–0.98)
5	69 906	4176	0.90 (0.87–0.94)
6–9	79 910	4853	0.93 (0.89–0.96)
		*P* for trend=0.0001	

Abbreviations: CI=confidence interval; HR=hazard ratio.

a Stratified by centre and sex, and adjusted for smoking status, duration of smoking, education, height, body mass index, total energy intake, physical activity and, for women, age at menarche, parity, menopausal status, oral contraceptive and hormone therapy use.

**Table 4:** Daily intake of foods groups considered in the Mediterranean diet score and associated hazard ratios for overall cancer

	**Mean±s.d.**	**Increment^a^**	**HR^b^ (95% CI)**
Overall score	4.4±1.7	2	0.96 (0.95–0.98)
Fruits and nuts (g per day)	247.3±197.2	200	0.98 (0.96–0.99)
Vegetables (g per day)	211.2±146.1	145	0.97 (0.95–0.98)
Legumes (g per day)	14.6±23.4	25	1.00 (0.99–1.02)
Cereals (g per day)	219.0±110.8	110	0.97 (0.95–0.98)
Dairy products (g per day)	326.7±235.2	235	1.01 (0.99–1.02)
Fish (g per day)	37.2±35.8	35	1.01 (0.99–1.02)
Meat (g per day)	98.7±6.3	60	1.02 (1.01–1.04)
Monounsaturated lipids (g per day)	30.9±13.8	15	1.01 (0.98–1.04)
Polyunsaturated lipids (g per day)	14.2±6.5	5	0.98 (0.97–1.00)
Saturated lipids (g per day)	31.5±13.0	15	1.01 (0.99–1.04)
Ratio of unsaturated to saturated lipids (g per day)	1.5±0.5	0.5	0.98 (0.96–0.99)
*Alcohol* c			
Light drinkers	1.9±2.2		1.03 (1.00–1.06)
Moderate drinkers	16.3±10.0		1.00
Heavy drinkers	51.8±25.4		1.12 (1.07–1.16)

Abbreviations: CI=confidence interval; HR=hazard ratio.

a The increment is approximately equal to the standard deviation (except for the Mediterranean diet score).

b Stratified by centre and sex, and adjusted for duration of smoking, smoking status, education, height, body mass index, total energy intake, physical activity and, for women, age at menarche, parity, menopausal status, oral contraceptive and hormone therapy use.

c Light drinkers: <10 g of ethanol per day for men and <5 g per day for women; moderate drinkers: 10 to <50 g per day for men and 5 to <25 g per day for women; heavy drinkers: ⩾50 g per day for men and ⩾25 g per day for women. HRs of cancer risk for category of alcohol consumption were calculated using moderate drinkers as the baseline category.

**Table 5:** HRs for all cancers associated with a two-point increment of the Mediterranean diet score for each category of tobacco smoking status

	**Cases**	**HR^a^ (95% CI)**
*All cancers*	30 731	0.96 (0.95–0.98)
Never smokers	13 787	0.98 (0.96–1.00)
Former smokers	8860	0.96 (0.93–0.98)
Current smokers	7439	0.94 (0.91–0.97)
	*P* for heterogeneity=0.07	
		
*Cancers known to be related to smoking* b ^,^ c	8000	0.92 (0.89–0.94)
Never smokers	2392	0.95 (0.90–0.99)
Former smokers	2490	0.93 (0.89–0.98)
Current smokers	3009	0.88 (0.84–0.92)
	*P* for heterogeneity=0.04	
		
*Cancers not known to be associated with smoking* c ^,^ d	11 218	0.98 (0.96–1.01)
Never smokers	5645	0.97 (0.94–1.01)
Former smokers	3205	0.99 (0.94–1.03)
Current smokers	2104	0.99 (0.94–1.04)
	*P* for heterogeneity=0.73	

Abbreviations: CI=confidence interval; HR=hazard ratio.

a Stratified by centre and sex, and adjusted for duration of smoking, smoking status, education, height, body mass index, total energy intake, physical activity, age at menarche, parity, menopausal status, oral contraceptive and hormone therapy use.

b Smoking-associated cancers include cancers of the lung, kidney, upper aero-digestive tract, stomach, pancreas, bladder, liver and colorectal.

c *P* for heterogeneity of smoking unrelated cancers *vs* smoking related cancers=0.0008

d Smoking-unrelated cancers include cancers of the breast, prostate, and endometrium.

## Appendix

Additional analyses were performed using a more quantitative Mediterranean diet score, based on the same nine dietary components: intake of fruits and nuts, vegetables, legumes, cereals, lipids, fish, dairy and meat products, and alcohol. The consumption of each component was log-transformed, country- and sex-specific means were calculated (means of log-transformed values approximate medians), and the distance to the mean was estimated for each component. This value was multiplied by 1 for components more consumed in Mediterranean countries (vegetables, legumes, fruits and nuts, cereals, fish, unsaturated-to-saturated lipid ratio), and −1 for those less consumed components (meat and dairy products). For alcohol intake, the score was calculated as *Z*−*m*/*s*, where *Z*=0 if the consumption *Y* falls in the ‘good’ interval (5 and <25 g per day for women, and 10 to <50 for men), *Z*=*Y*−min if *Y*<min, and *Z*=max−*Y* if *Y*>max; *m*≡*Z¯, s*≡*sd* (*z*). These components were then summed up to obtain an overall score. A high overall Mediterranean diet score shows a high adherence to the Mediterranean dietary pattern and the opposite for a low score.
